# Physical activity and genetic predisposition to obesity in a multiethnic longitudinal study

**DOI:** 10.1038/srep18672

**Published:** 2016-01-04

**Authors:** Hudson Reddon, Hertzel C. Gerstein, James C. Engert, Viswanathan Mohan, Jackie Bosch, Dipika Desai, Swneke D. Bailey, Rafael Diaz, Salim Yusuf, Sonia S. Anand, David Meyre

**Affiliations:** 1 Department of Clinical Epidemiology and Biostatistics, McMaster University, Hamilton, Ontario, Canada; 2 Population Health Research Institute, McMaster University and Hamilton Health Sciences, Hamilton General Hospital, Hamilton, Ontario, Canada; 3 Department of Medicine, McMaster University, Hamilton, Ontario, Canada; 4 Departments of Medicine and Human Genetics, McGill University, Montreal, QC, Canada; 5 Madras Diabetes Research Foundation, Chennai, India; 6 ECLA—Academic Research Organization, Rosario, Argentina; 7 Department of Pathology and Molecular Medicine, McMaster University, Hamilton, Ontario, Canada

## Abstract

Physical activity (PA) has been shown to reduce the impact of *FTO* variation and obesity genetic risk scores (GRS) on BMI. We examined this interaction using a quantitative measure of PA and two adiposity indexes in a longitudinal multi-ethnic study. We analyzed the impact of PA on the association between 14 obesity predisposing variants (analyzed independently and as a GRS) and baseline/follow-up obesity measures in the multi-ethnic prospective cohort EpiDREAM (17423 participants from six ethnic groups). PA was analyzed using basic (low-moderate-high) and quantitative measures (metabolic equivalents (METS)), while BMI and the body adiposity index (BAI) were used to measure obesity. Increased PA was associated with decreased BMI/BAI at baseline/follow-up. *FTO* rs1421085, *CDKAL1* rs2206734, *TNNl3K* rs1514176, *GIPR* rs11671664 and the GRS were associated with obesity measures at baseline and/or follow-up. Risk alleles of three SNPs displayed nominal associations with increased (*NTRK2* rs1211166, *BDNF* rs1401635) or decreased (*NPC1* rs1805081) basic PA score independently of BMI/BAI. Both basic and quantitative PA measures attenuated the association between *FTO* rs1421085 risk allele and BMI/BAI at baseline and follow-up. Our results show that physical activity can blunt the genetic effect of *FTO* rs1421085 on adiposity by 36–75% in a longitudinal multi-ethnic cohort.

Obesity has become a global epidemic^1^ and is a known risk factor for a number of adverse health outcomes, including psychological disturbance, osteoarthritis, type 2 diabetes, hypertension, cardiovascular disease, cancer and ultimately 8–13 years shorter life expectancy in its more severe forms^2^,^3^. Since the rising prevalence of obesity has been attributed primarily to changes in environmental exposures, such as excessive energy intake, sedentary lifestyle and sleep debt, among others^4^, recent research has focused on preventative strategies to control the obesity epidemic^5^.

Despite the global impact of these environmental changes, obesity appears to manifest preferentially in genetically predisposed individuals, and a high level of inter-individual variation has been observed among exposed populations^6^. Current evidence has shown that heritability estimates for obesity-related traits can be modulated by lifestyle factors such as physical activity (PA)^7^. Significant gene-environment interactions (GEI) between *FTO* intron 1 variation and PA have been consistently found in 16 independent cross-sectional and intervention studies performed with children and adult populations of European, East Asian and African ancestry^8^,^9^,^10^. A recent meta-analysis in 111 421 subjects of European ancestry confirmed a significant PA x genetic risk score (GRS) interaction using 12 obesity predisposing SNPs and showed that this interaction was more apparent in subjects living in North America^11^.

Although these data provide convincing evidence of an interaction between genetic predisposition to obesity and PA, we identified several important limitations of the existing literature. First, almost all current gene x PA interaction studies in the obesity field have been conducted using cross sectional designs, although there are exceptions^12^. Strengths of the cohort design include the ability to study temporal relationships, which strengthens the confidence in statistical associations^13^,^14^. Second, most GEI analyses of PA have used imprecise self-report measurements of PA due to concerns regarding cost, feasibility and participant burden^15^ and only one study has examined the impact of a quantitative metabolic equivalent (MET) score^16^. Third, nearly all GEI studies of obesity have used BMI as the outcome measure of obesity, although the validity of the BMI can be compromised by different body compositions (e.g. lean vs. fat mass)^17^. Fourth, GEI studies have mainly been performed in European populations, but the transferability of the conclusions of these studies to other ethnic backgrounds remains unclear^18^,^19^. Although a previous meta-analysis analyzed multiple ethnic groups, the sample was over 95% European^18^. This prompted us to assess (1) the association between PA behavior and obesity and (2) the interaction between 14 obesity predisposing variants (analyzed independently and as a GRS) and PA on obesity-related traits in the multi-ethnic longitudinal cohort EpiDREAM. We used a quantitative measure of energy expenditure (Metabolic Equivalent Score, MET score)^20^ and the recently validated body adiposity index (BAI)^21^. The BAI may provide new insight into this interaction due to its strong correlation (r = 0.85) with body adiposity (as measured by dual x-ray absorptiometry)^21^.

## Materials and Methods

### Study Participants

The data for this investigation were collected through a prospective cohort of participants at risk for type 2 diabetes (T2D), which has been described in detail previously^22^,^23^. Briefly, EpiDREAM enrolled a total of 24 872 individuals recruited from 21 countries who were screened for eligibility to enter the DREAM clinical trial^22^. Individuals who were identified as at risk for type 2 diabetes based on family history, ethnicity and abdominal adiposity were screened using a 75-gram oral glucose tolerance test (OGTT). A subset of 1960 DREAM participants received rosiglitazone over follow-up. All participants were between the ages of 18–85 years and were screened between July 2001 and August 2003. We focused on 17 423 subjects from six ethnic groups (South Asian, East Asian, European, African, Latin American, Native North American) having both phenotypic and 50 K gene-centric array information in the EpiDREAM study (Supplementary Figure 1). Self-reported ethnicity has been verified in the 17 423 individuals using the eigensoft software (http://genepath.med.harvard.edu/~reich/Software.htm) and 40 individuals were reclassified. The first 10 components from this principal components analysis were retained to adjust for population stratification (Supplementary Figure 2).

Of the 17 423 participants at baseline, our follow-up analyses included 9 228 participants with complete genotype and phenotype data. Interim and final visits with participants occurred 12–24 months and 36–48 months (median follow-up 3.3 years) after their screening visit, respectively. Participants who were not able to complete in-person clinic visits were contacted through telephone or mail^23^. The EpiDREAM study has been approved by local ethics committees and the study methodology was carried out in accordance with the approved guidelines. Informed consent was obtained from each subject before participating in the study, in accordance with the Declaration of Helsinki.

### Genotyping

Buffy coats for DNA extraction have been collected in 19 197 participants of the EpiDREAM study (Supplementary Figure 1). DNA has been extracted by the Gentra System. Genotyping was performed using the Illumina CVD bead chip microarray ITMAT Broad Care (IBC) array^24^. Genotyping was performed at the McGill University and Genome Quebec Innovation Centre using the Illumina Bead Studio genotyping module, version 3.2. We established a list of SNPs that reached genome-wide significance (P < 5 × 10^−8^) with BMI or binary obesity status in populations of European ancestry. We used three different strategies to optimize the SNP selection procedure using a key word search (e.g. BMI) on i) the National Human Genome Research Institute (NHGRI) GWAS Catalog (www.genome.gov/gwastudies/) ii) the HuGE Navigator GWAS Integrator (www.hugenavigator.net/HuGENavigator/gWAHitStartPage.do) iii) the PubMed database (www.ncbi.nlm.nih.gov/pubmed). Using this strategy, we ended-up with a list of 72 independent SNPs associated with obesity-related traits. From this list, 14 SNPs were available on versions 1 and 2 of the IBC 50 K SNP array (Supplementary Table 1). The SNPs selected showed no significant (P < 10^−6^) deviation from Hardy-Weinberg Equilibrium (HWE) in the six ethnic groups. The call rate for each of the 14 SNPs was between 99.8–100% (Supplementary Table 1).

### Phenotyping

In addition to the OGTT, participants completed a questionnaire that included demographic data, medical history and PA behaviors at baseline and follow-up. Anthropometric measurements including height, weight, waist and hip circumference were performed using a standardized protocol^22^. Height (m), weight and hip circumference (HC) (cm) were measured by trained medical staff. Standing height was measured to the nearest 0.1 cm and weight was measured to the nearest 0.1 kg in light clothing. Hip circumference was measured in duplicate at the level of the greater trochanters using a non-flexible tape measure with an attached spring balance with a mass of 750 g. Averages of the two measures were used in all analyses. Body mass index (BMI) was calculated as weight in kilograms divided by height in meters (m) squared. We used Body Adiposity Index (BAI), which estimates body adiposity percentage directly based on height and hip circumference^25^. Specifically, BAI = [(hip circumference)/((height)^(1.5)^) − 18], with hip circumference and height being expressed in centimeters and meters, respectively^25^.

The 2003 ADA criteria were used to classify participants as having normal glucose tolerance (NGT), impaired fasting glucose (IFG), impaired glucose tolerance (IGT), or T2D at baseline, as confirmed by an oral glucose tolerance test. Normoglycemia was defined as a fasting plasma glucose < 5.6 mmol/L, IFG was defined as a fasting plasma glucose of 5.6 to 6.9 mmol/L, IGT was defined as a fasting plasma glucose below 7.0 mmol/L and a 2-h glucose between 7.8 and 11.0 mmol/L, and diabetes was defined if either the fasting plasma glucose was ≥ 7.0 mmol/L or the 2-h glucose was ≥ 11.1 mmol/L^26^. IFG and IGT were collapsed into one category and these three groups (normoglycemia, IFG/IGT, diabetes) comprised the glycemic status variable.

PA measures were based on self-reported time of participation in 41 different physical activities (Supplementary Table 2). A quantitative measure of energy expenditure (Metabolic Equivalent score, MET score) was derived using this information and the updated compendium of physical activities^20^. A MET score was assigned to each activity based on the energy cost of that activity. The energy expended on each leisure time and work-related activity was estimated by multiplying the hours/week of participation by the corresponding MET value. These measures were summed across all activities to provide an overall estimate of energy expenditure represented as MET minutes per week. This quantitative measure was compared to a brief, self-reported, categorical measure based on PA participation during leisure time and at work. Participants rated their PA level at work and during leisure on a scale of one to three to form the basic PA score (1 = sedentary, 2 = moderately active, 3 = very active).

### Statistical Analyses

Statistical analyses were performed using SPSS (version 20, New York, USA, IBM Corporation) and the power of our study was calculated using QUANTO (version 1.2.4, University of Southern California, Los Angeles, CA, USA). Single SNP analyses were performed under the additive model, and the obesity risk alleles previously identified for each of the 14 SNPs in literature were used as the risk allele. General linear models (GLM) were used to examine (1) the association between energy expenditure (METS)/basic PA score at baseline and BMI/BAI at baseline and follow-up (2) the association between 14 obesity predisposing gene variants (analyzed independently and as a GRS) and BMI/BAI at baseline and follow-up. These tests were adjusted for covariates including, sex, age, ethnicity and glycemic status. General linear models were used, with or without the inclusion of a SNP x PA or a GRS x PA interaction term. Since previous studies have demonstrated a compelling association between each of the SNPs analyzed and obesity traits, the effect of the SNPs on BMI/BAI was considered a confirmatory step. Therefore, all SNPs reaching a nominal level of association with BMI/BAI (*P* < 0.05) were considered significant and followed up for interaction analysis. These tests were also adjusted for covariates including, sex, age, ethnicity and glycemic status. All analyses with BMI/BAI follow-up or change as the dependent variable were adjusted for rosiglitazone use in addition to the aforementioned variables. A natural log transformation was used successfully to correct the positive skew of the MET score data. Ordinal logistic regression was used to analyze the impact of the 14 obesity predisposing SNPs on the basic PA score (adjusted for sex, age, ethnicity, glycemic status and BMI). This test was performed since a correlation between the genetic variant and environmental exposure can bias the estimates of the main genetic effect and the gene-environment interaction^27^. Previous examples of these effects have been observed in cancer research, where variants on 15q25 have been linked to both smoking behaviors and lung cancer^28^. The genetic predisposition score was calculated by summing the alleles of the 14 obesity predisposing SNPs so that the score could range from 0 to 28. Since weighting has been shown to have no major impact on the effect of a GRS^29^, an unweighted GRS was used. We performed imputations for missing genotypic values as previously described^30^ using the mean number of predisposing obesity alleles in successfully genotyped individuals. This procedure was performed separately in each ethnic group. Individuals with more than one out of 14 missing genotype were not included in the genetic risk score calculation. Although multiple SNPs included in the gene score are within the same gene, our linkage disequilibrium analyses of this cohort indicated that it is appropriate to include all 14 SNPs in the genetic risk score (r^2^ < 0.25, Supplementary Table 3). Two-tailed P-values are presented in this manuscript and *P* < 0.05 were considered as nominally significant. After applying a Bonferroni’s correction for multiple testing, a *P* < 0.00025 (0.05/200) was considered as significant.

## Results

### Characteristics of the studied cohort

The clinical and anthropometric characteristics of the EpiDREAM study are summarized in Table 1. The mean age of participants was 52.7 years and the ethnic distribution of the cohort was 53.9% European, 18.9% Latino, 15.8% South Asian, 7.2% African, 2.9% Native American, 1.3% East Asian. The individuals in this analysis represented 17 of the original 21 countries from which recruitment took place (Supplementary Table 4). At baseline, a mean BMI of 30.2 (SD = 6.22) kg/m^2^ and a mean BAI of 33.0 (SD = 7.49) were observed and the mean energy expenditure was 320.50 MET-minutes/week (SD = 409.20). For the present analyses, we focused on 17 423 participants at baseline and 9 228 at follow-up who had complete genotype and phenotype data. The median time between the baseline screening visit and final contact was 3.3 years. After follow-up, the mean BMI and BAI were 30.32 (SD = 5.79) and 33.78 (SD = 7.59), respectively, and the mean energy expenditure appeared to decrease slightly to 301.50 MET-minutes/week (SD = 368.04).

### Effect of Physical Activity on BMI/BAI

At baseline, the quantitative MET score was significantly associated with both decreased BMI and BAI (Table 2). Congruently, the basic PA score (low – moderate – high PA) was significantly associated with lower baseline BMI and BAI. Similar associations were found between the baseline MET score and BMI and BAI at follow-up. The baseline basic PA score was also associated with decreased follow-up BMI and BAI. The baseline MET score was not associated with BMI change, or BAI change. The basic PA score was nominally associated with decreased BMI change and BAI change over follow-up.

### Effect of SNPs/GRS on Physical Activity

We first investigated the association of 14 obesity predisposing SNPs and corresponding GRS on basic PA score adjusting for sex, age, ethnicity, glycemic status and BMI (Table 3). We observed a nominal association between three of these SNPs and the basic PA score: *NTRK2* rs1211166, *BDNF* rs1401635 and *NPC1* rs1805081. The association between the obesity risk GRS and the basic PA score was not significant. When adjusting for BAI rather than BMI the same three SNPs remained nominally associated with the basic PA score with a consistent direction of effect (Supplementary Table 5).

None of the 14 SNPs displayed a significant association with the MET score after adjustment for sex, age, ethnicity, glycemic status and BMI (Table 3). The association between the obesity risk GRS and the MET score was also non-significant. Similar results were found when adjusting for BAI rather than BMI (Supplementary Table 5).

Of the 14 SNPs analyzed, only one (*NTRK2* rs1211166) showed a nominal association with change in the basic PA score (Supplementary Table 6). None of the 14 SNPs displayed a significant association with a change in the MET score. The obesity risk GRS was not associated with change in the basic PA score or change in the MET score (Supplementary Table 6).

### Effect of SNPs/GRS on BMI/BAI

At baseline, the obesity risk alleles of four SNPs were associated increased with BMI and BAI. *FTO* rs1421085 and *CDKAL1* rs2206734 were significantly associated with greater BMI/BAI, while *TNNI3K* rs1514176 and *GIPR* rs11671664 were nominally associated with increased BMI/BAI (Table 4). At baseline, the GRS was significantly associated with greater BMI and BAI.

After follow-up, three SNPs (*FTO* rs1421085, *TNNI3K*, rs1514176, *GIPR* 11671664) and the GRS were associated with increased BMI and BAI (Table 4). *CDKAL1* rs2206734 displayed a nominal association with reduced BMI and BAI change. The GRS was not associated with BMI or BAI change.

### Interaction Analyses

Interaction tests with PA were restricted to the subset of SNPs/GRS displaying a nominal or significant association with BMI/BAI at baseline and/or at follow-up. At baseline, the MET score modified the effect of the *FTO* risk allele on BMI and BAI (Table 5). Each additional *FTO* risk allele (C) was associated with a (1) BMI increase of 0.60 kg/m^2^ (P = 1.1 × 10^−4^), BAI increase of 0.45 (P = 5.7 × 10^−3^) in the lowest MET score tertile and (2) BMI increase of 0.26 kg/m^2^ (P = 0.05), BAI increase of 0.20 (P = 0.15) in the highest MET score tertile. This indicates that the effect of *FTO* rs1421085 on BMI and BAI can be reduced by 57% and 56% respectively through PA.

**Figure 1:**
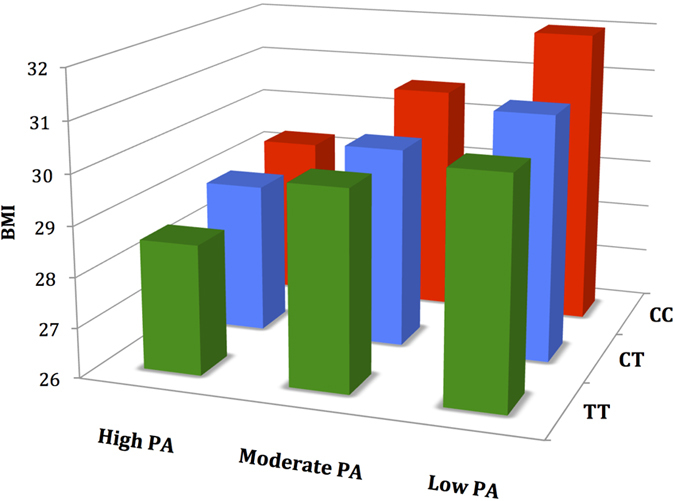
Mean baseline BMI values stratified by physical activity level (PA) and *FTO* rs1421085 genotype.

The basic physical score also modified the effect of the *FTO* risk allele on BMI and BAI at baseline (Table 5, Fig. 1). Each additional obesity risk allele (C) was associated with a (1) BMI increase of 0.71 kg/m^2^ (P = 1.4 × 10^−7^), BAI increase of 0.62 (P = 2.1 × 10^−5^) in the inactive group and (2) BMI increase of 0.35 kg/m^2^ (P = 0.03), BAI increase of 0.40 (P = 0.02) in the active group. This indicates that PA is associated with a 36–51% decrease in the effect of *FTO* rs1421085 on obesity measures at baseline. We also conducted a sensitivity analysis to analyze the BPA x *FTO* rs1421085 interaction for baseline BMI in only non-diabetic patients, and found similar results (β = −0.40, 95% CI = −0.62 to −0.19, P = 2.4 × 10^−4^).

The basic PA score interacted with *FTO* rs1421085 in modulating BMI and BAI at follow-up. Each additional obesity risk allele (C) was associated with a (1) BMI increase of 0.72 kg/m^2^ (P = 1.1 × 10^−5^), BAI increase of 0.75 (P = 3.4 × 10^−4^) in the inactive group and (2) BMI increase of 0.19 kg/m^2^ (P = 0.39), BAI increase of 0.19 (P = 0.44) in the active group. This corresponds to a 74–75% decrease in the effect of *FTO* rs1421085 on BMI/BAI at follow-up.

No significant interactions were observed between *TNNI3K* rs1514176, *CDKAL1* rs2206734, *GIPR* rs11671664 or the obesity risk GRS and the basic PA/MET score on BMI/BAI at baseline, follow-up or change.

Given that Ahmad *et al.*^11^ reported the *FTO* x PA interaction to be 10-fold larger in North American compared to European cohorts, we also analyzed a 3-way interaction (*FTO* x PA x North American residence) among the subgroup of European participants to follow-up this finding. Although the 3-way interaction was not significant (β = −0.01, 95% CI = −0.03 to 2.8 × 10^−3^, P = 0.10), we acknowledge that our statistical power to detect 3-way interactions in this subgroup was limited and this association warrants further investigation.

## Discussion

We observed significant GEI between *FTO* rs1421085 and PA at baseline and at follow-up in an international multiethnic population using both basic and quantitative assessments of PA. Although the interactions were nominally significant, the Bonferroni correction we applied is overly conservative, particularly when testing highly correlated outcomes^31^ such as BMI/BAI, basic PA/MET score, and we have confidence in our results for several reasons. First, the power to detect GEI at the nominal level of association (P < 0.05) is adequate at baseline, although our power estimations for the Bonferroni corrected threshold are moderate (see Supplementary Figures 3–6). Second, twin studies have shown that PA can substantially reduce the influence of genetic factors on BMI in adults^7^. Third, the interaction between *FTO* and PA has been demonstrated in several cross-sectional studies, and currently represents the most robust example of GEI in the field of genetic epidemiology^8^,^9^,^10^,^11^,^18^. Fourth, there is a plausible underlying biological process to substantiate this association. *FTO* is a nucleic acid demethylase and *FTO* intron 1 variation is associated with different methylation profiles and BMI variance^32^,^33^,^34^. Since methylation of DNA is sensitive to environmental changes (e.g. PA and diet) there is a strong biological rationale to identify GEI with *FTO* as previously reported^8^,^35^. Two studies have shown that PA can change the methylation and mRNA expression pattern of genes, including *FTO*, in both muscle and adipose tissue^34^,^36^. A more recent analysis demonstrated that variation at the *FTO* locus represses mitochondrial thermogenesis in adipocyte precursor cells and causes a shift from energy dissipating beige (brite) to energy-storing white adipocytes, which is accompanied by increased lipid storage and weight gain^37^. Exercise studies in humans and mouse models indicate that exercise training increases the expression of the brown adipocyte marker uncoupling protein (UCP1) in both visceral and subcutaneous white adipose tissue (WAT)^38^. These changes are associated with increases in brown-like adipocytes (browning or beiging), particularly in subcutaneous WAT^38^. The combined role of FTO and physical activity in obesity and adipocyte browning, in conjunction with epigenetic mechanisms, strengthen the biological rationale and confidence in the statistical interaction^39^.

Given the growing consensus that food intake may be the main driver of the obesity epidemic^40^, it is important to note that both PA measures displayed significant associations with both adiposity measures at baseline and at follow-up. This indicates that PA can influence obesity, despite the broad range of lifestyles among the participants. The value of PA for managing obesity has been recognized in a recent analysis of the National Health and Nutrition Examination Survey (NHANES) cohort from 1988–2010, which found that PA had a larger impact on BMI and waist circumference trends than calorie intake^41^. Our cross sectional analyses indicate that one hour of jogging or swimming (8.0 MET activities) per week was associated with approximately a 0.5 kg/m^2^ decrease in BMI. Together, these data challenge the idea of attributing the obesity epidemic mainly to excessive caloric intake^40^ and support the universal value of PA to maintain a healthy body weight^41^.

Although the MET score provides a more comprehensive assessment of PA participation, only 11 015 (63%) participants completed the assessment of the MET score, compared to the 17 407 (99%) participants who completed the basic PA score. The loss of power induced by the smaller sample size may have been compensated for by the added precision of the MET score. Simulations of GEI have shown that a sample of about 2 000 participants with precisely measured environmental exposure and outcome data are needed to detect a GEI of large magnitude (a doubling of the genetic risk estimate in the exposed group compared to the unexposed group) with reasonable power (95% power, P = 1 × 10^−4^)^42^. With less precise measurement of environmental exposure, the sample size requirement can increase to 100 000 participants to detect the same interaction with comparable power^42^,^43^. Using a brief PA assessment may be the best compromise to balance the sample size requirements with the need for sufficient precision, as recently suggested by Peters *et al.*^44^.

Measuring body fat content data is less feasible in large sample sizes for GEI studies. Only two small GEI studies (N < 800) have used direct body fat content measures (DEXA and underwater weighing)^45^,^46^ and the meta-analysis of the PA x *FTO* interaction which analyzed bioelectrical impedance was over 95% Europeans^16^. To our knowledge, this is the first large-scale study to report an interaction between PA and *FTO* rs1421085 using the BAI. Since assessing body fat content is financially prohibitive in large-scale analyses, the BAI may be an acceptable method to complement the widely used BMI measure to assess adiposity in GEI studies.

Assessing the impact of the 14 SNPs on PA identified some interesting nominal associations. Despite being an obesity predisposing gene, *BDNF* may be related to increased PA levels in our cohort. While this appears contradictory, the loss of one functional copy of *BDNF* gene has been associated with Mendelian obesity, cognitive impairment and hyperactivity in both humans^47^ and rodents^48^. It has been proposed that increased PA may be a behavioral response to compensate for their proclivity to gain weight^49^. Alternatively, these SNPs have been hypothesized to be a remnant of our hunter-gatherer past, and may have been positively selected since they promote an “active-foraging” phenotype that induces a preference for energy-dense foods and the physical disposition to attain those foods^49^. Together, this food seeking behaviour and overactive disposition represent a “Peppy-Thrifty Genotype.” In contrast, *NPC1* variation appears to contribute to sedentary behavior and may complement the thrifty genotype hypothesis^50^,^51^. Genetic variants increasing food intake and decreasing PA may have been positively selected based on their parallel effects on energy balance^51^. The contrasting effects of these obesity predisposing SNPs on PA may account for the challenges in substantiating the lazy thrifty genotype hypothesis^52^. Although there appears to be some shared genetic correlation between obesity and PA, additional studies are necessary to confirm our nominally significant associations and to clarify the impact of additional obesity predisposing genes on PA.

Limitations of this study include the multi-ethnic composition of the EpiDREAM cohort may have added significant heterogeneity in the analyses, especially as important PA differences are observed in different ethnic backgrounds^53^. Although, the MET score was calculated with more objective criteria of PA participation, recalling participation in 41 different activities introduces a source of error and or recall bias. Since most of the obesity predisposing SNPs selected in the study were originally identified in European populations, they may not be ideal proxies for the causal SNP in other ethnic groups. We are aware that the 14 SNPs analyzed only represent a subset of the current list of validated obesity SNPs. Lastly, the EpiDREAM population (participants identified for hyperglycemia risk) is not representative of the general population, and the participants missing from the follow-up analysis may have created a systematic bias in our sample. However, this bias may not have influenced our results since no significant differences in BMI (P = 0.25) or BAI (P = 0.21) were observed among those who completed follow-up and those that did not.

Strengths of this analysis include the precise MET score, complementing the BMI with the recently developed BAI, the prospective cohort design and the multi-ethnic sample.

In summary, we identified an interaction between the *FTO* SNP rs1421085 and PA in a prospective cohort of six ethnic groups from 17 countries. While this has been demonstrated previously, this is the first study to analyze this interaction prospectively using a quantitative measure of PA while comparing the recently developed BAI and BMI. Analyzing the impact of obesity predisposing SNPs on PA revealed novel associations, although further study is needed to confirm these effects. These findings suggest that obesity prevention programs emphasizing vigorous PA for genetically at risk subgroups may be a valuable contribution to the global fight against obesity.

## Additional Information

**How to cite this article**: Reddon, H. *et al.* Physical activity and genetic predisposition to obesity in a multiethnic longitudinal study. *Sci. Rep.* **6**, 18672; doi: 10.1038/srep18672 (2016).

## Supplementary Material

Supplementary Information

## Figures and Tables

**Table 1:** Baseline characteristics by physical activity status in EpiDREAM study.

	Category	PA-Low	PA-Moderate	PA-High	All	p-value
Total at baseline N(%)		4727 (27.1%)	10529 (60.4%)	2151 (12.3%)	17407 (100%)	
Gender N(%)	Male	1498 (31.6%)	4088 (38.8%)	1213 (56.4%)	6799 (39.1%)	3.8 × 10^−83^
	Female	3229 (68.3%)	6441 (61.2%)	938 (45.6%)	10608 (60.9%)	
aAge (years)		51.24 ± 12.13	53.17 ± 11.05	53.25 ± 10.97	52.66 ± 11.38	1.5 × 10^−22^
Glycemic status N (%)	Normal	2138 (45.2%)	4384 (41.6%)	921 (42.8%)	7443 (42.7%)	1.8 × 10^−4^
	IFG	1821 (38.5%)	4614 (43.8%)	961 (44.7%)	7396 (42.5%)	6.6 × 10^−10^
	Diabetes	768 (16.3%)	1531 (14.6%)	269 (12.5%)	2568 (14.8%)	1.7 × 10^−4^
aMETS at baseline		165.15 ± 220.80 (2038)	302.98 ± 353.17 (7290)	584.82 ± 629.36 (1682)	320.54 ± 409.20 (11010)	2.2 × 10^−23^
aBMI at baseline (kg/m^2^)		30.90 ± 7.07 (4726)	30.09 ± 5.96 (10527)	28.89 ± 5.17 (2153)	30.16 ± 6.22 (17407)	3.2 × 10^−35^
aBMI at follow-up (kg/m^2^)		30.81 ± 6.23 (2429)	30.35 ± 5.67 (5706)	29.03 ± 5.11 (1070)	30.32 ± 5.79 (9205)	2.1 × 10^−16^
aBMI Change (kg/m^2^)		0.08 ± 2.85 (2427)	0.25 ± 2.41 (5704)	0.28 ± 2.12 (1070)	0.21 ± 2.50 (9201)	0.22
aBAI at baseline		35.19 ± 8.08 (4716)	32.65 ± 7.20 (10500)	29.91 ± 6.02 (2138)	33.00 ± 7.49 (17354)	3.4 × 10^−75^
aBAI at follow-up		35.51 ± 8.19 (2312)	33.67 ± 7.37 (5487)	30.48 ± 6.07 (1033)	33.78 ± 7.59 (8832)	2.7 × 10^−55^
aBAI Change		0.09 ± 3.92 (2309)	0.56 ± 3.65 (5482)	0.52 ± 3.28 (1032)	0.43 ± 3.69 (8823)	5.0 × 10^−43^
Ethnic groups N(%)	South Asian	1527 (32.3%)	1122 (10.7%)	111 (5.2%)	2760 (15.8%)	8.4 × 10^−15^
	East Asian	34 (0.7%)	154 (1.5%)	37 (1.7%)	225 (1.3%)	
	European	1689 (35.7%)	6091 (57.8%)	1601 (74.4%)	9381 (53.9%)	
	African	362 (7.7%)	793 (7.5%)	94 (4.4%)	1249 (7.2%)	
	Latino American	1002 (21.2%)	2057 (19.5%)	233 (10.8%)	3292 (18.9%)	
	Native-North American	113 (2.4%)	312 (3.0%)	75 (3.5%)	500 (2.9%)	

Notes: BMI: body mass index, BAI: body adiposity index, SD: standard deviation, N: sample size, PA: physical activity

^a^Data are presented as mean ± SD (N)

**Table 2:** Effect of physical activity on obesity outcomes.

	Outcome	β	95% CI	P-value
Effect of MET score on BMI/BAI (adjusted for gender, age, ethnicity and glycemic status)				
Baseline	BMI	−0.54	−0.63 to −0.45	1.2 × 10^−30^
	BAI	−0.59	−0.69 to −0.49	8.6 × 10^−32^
Follow-up	BMI	−0.45	−0.57 to −0.34	1.1 × 10^−13^
	BAI	−0.59	−0.73 to −0.45	3.6 × 10^−16^
Change	BMI	−0.05	−4.1 × 10^−4^ to 0.11	0.26
	BAI	0.07	−0.01 to 0.15	0.07
Effect of basic physical activity score on BMI/BAI (adjusted for gender, age, ethnicity and glycemic status)				
Baseline	BMI	−1.59	−1.74 to −1.44	5.4 × 10^−59^
	BAI	−1.78	−1.94 to −1.62	5.4 × 10^−61^
Follow-up	BMI	−1.35	−1.54 to −1.15	1.2 × 10^−41^
	BAI	−1.62	−1.86 to −1.39	7.3 × 10^−42^
Change	BMI	−0.12	−0.22 to −0.04	0.01
	BAI	−0.14	−0.28 to −0.01	0.04

**Table 3:** Effect of SNPs/GRS on physical activity measures.

SNP	Gene	OR (95% CI)^a^	P-Value^a^	β (95% CI)^b^	P-Value^b^
rs1514176	*TNNI3K*	1.02 (0.97 to 1.06)	0.44	−0.01 (−0.04 to 0.03)	0.74
rs6235	*PCSK1*	0.95 (0.90 to 1.01)	0.40	0.01 (−0.03 to 0.04)	0.81
rs6232	*PCSK*	1.03 (0.92 to 1.15)	0.67	−0.01 (−0.09 to 0.07)	0.76
rs2206734	*CDKAL1*	0.99 (0.94 to 1.05)	0.82	−0.01 (−0.05 to 0.02)	0.47
rs2272903	*TFAP2B*	1.03 (0.97 to 1.09)	0.40	0.01 (−0.04 to 0.05)	0.76
rs1211166	*NTRK2*	1.07 (1.01 to 1.13)	0.02	3.0 × 10^−3^ (−0.04 to 0.04)	0.88
rs6265	*BDNF*	1.05 (0.99 to 1.12)	0.09	0.04 (−0.01 to 0.08)	0.10
rs1401635	*BDNF*	1.08 (1.03 to 1.14)	2.7 × 10^−3^	−0.01 (−0.05 to 0.02)	0.47
rs997295	*MAP2K5*	1.04 (0.99 to 1.09)	0.08	3.9 × 10^−3^ (−0.03 to 0.04)	0.81
rs7203521	*FTO*	1.02 (0.92 to 1.07)	0.30	−0.01 (−0.04 to 0.02)	0.61
rs1421085	*FTO*	0.96 (0.98 to 1.01)	0.13	0.01 (−0.02 to 0.05)	0.45
rs1805081	*NPC1*	0.92 (0.88 to 0.97)	6.2 × 10^−4^	−0.01 (−0.04 to 0.03)	0.64
rs2075650	*APOE*	0.96 (0.90 to 1.02)	0.20	−0.03 (−0.07 to 0.02)	0.29
rs11671664	*GIPR*	1.05 (0.98 to 1.12)	0.21	0.01 (−0.04 to 0.06)	0.79
	GRS	1.01 (0.99 to 1.02)	0.15	−3.5 × 10^−4^ (−0.01 to 0.01)	0.94

Notes: GRS = genetic risk score, all analyses were adjusted for sex, age, ethnicity, glycemic status and BMI.

^a^indicates analyses with the basic physical activity score as the dependent variable.

^b^indicates analyses with the MET score as the dependent variable.

**Table 4:** Effect of SNPs/GRS on obesity measures.

SNP	gene	BMI Baseline		BMI Follow-up		BMI Change	
		β (95% CI)	P-value	β (95% CI)	P-value	β (95% CI)	P-value
rs1514176	*TNNI3K*	0.19 (0.07 to 0.32)	2.2 × 10^−3^	0.17 (0.01 to 0.33)	0.04	−0.03 (−0.11 to 0.04)	0.38
rs6235	*PCSK1*	0.04 (−0.11 to 0.19)	0.62	4.4 × 10^−3^ (−0.19 to 0.19)	0.96	0.02 (−0.07 to 0.10)	0.74
rs6232	*PCSK*	0.04 (−0.27 to 0.36)	0.78	0.09 (−0.33 to 0.50)	0.68	−0.06 (−0.25 to 0.13)	0.52
rs2206734	*CDKAL1*	0.28 (0.13 to 0.44)	2.2 × 10^−4^	−0.01 (−0.21 to 0.18)	0.90	−0.15 (−0.24 to −0.06)	1.4 × 10^−3^
rs2272903	*TFAP2B*	0.11 (−0.07 to 0.28)	0.24	0.14 (−0.08 to 0.37)	0.21	−0.06 (−0.17 to 0.04)	0.23
rs1211166	*NTRK2*	0.05 (−0.10 to 0.20)	0.50	0.04 (−0.15 to 0.24)	0.67	−0.01 (−0.09 to 0.08)	0.91
rs6265	*BDNF*	0.10 (−0.07 to 0.27)	0.24	−0.02 (−0.24 to 0.19)	0.85	0.02 (−0.08 to 0.11)	0.77
rs1401635	*BDNF*	0.09 (−0.05 to 0.23)	0.21	0.11 (−0.07 to 0.30)	0.23	0.03 (−0.06 to 0.11)	0.51
rs997295	*MAP2K5*	0.05 (−0.08 to 0.17)	0.47	0.01 (−0.15 to 0.17)	0.92	0.01 (−0.07 to 0.08)	0.85
rs7203521	*FTO*	0.04 (−0.09 to 0.17)	0.55	−0.03 (−0.1519 to 0.14)	0.75	0.02 (−0.05 to 0.10)	0.58
rs1421085	*FTO*	0.49 (0.36 to 0.62)	3.7 × 10^−13^	0.49 (0.32 to 0.65)	1.7 × 10^−8^	−0.01 (−0.09 to 0.07)	0.80
rs1805081	*NPC1*	0.07 (−0.07 to 0.20)	0.31	0.09 (−0.08 to 0.26)	0.31	−1.4 × 10^−4^ (−0.08 to 0.08)	0.99
rs2075650	*APOE*	0.08 (−0.10 to 0.26)	0.38	−3.1 × 10^−3^ (−0.24 to 0.23)	0.98	−0.03 (−0.13 to 0.08)	0.64
rs11671664	*GIPR*	0.28 (0.09 to 0.48)	5.1 × 10^−3^	0.27 (0.02 to 0.52)	0.04	−0.01 (−0.13 to 0.11)	0.87
rs1514176	*TNNI3K*	0.14 (0.01 to 0.28)	0.04	0.19 (0.03 to 0.38)	0.04	−0.07 (−0.18 to 0.04)	0.22
rs6235	*PCSK1*	−0.01 (−0.17 to 0.15)	0.90	0.11 (−0.12 to 0.34)	0.37	0.04 (−0.09 to 0.18)	0.51
rs6232	*PCSK*	0.13 (−0.22 to 0.47)	0.46	0.20 (−0.31 to 0.70)	0.45	−0.14 (−0.43 to 0.15)	0.35
rs2206734	*CDKAL1*	0.34 (0.18 to 0.51)	4.0 × 10^−5^	0.6 (−0.18 to 0.30)	0.61	−0.19 (−0.32 to −0.05)	6.5 × 10^−3^
rs2272903	*TFAP2B*	0.20 (−0.01 to 0.39)	0.06	0.16 (−0.12 to 0.44)	0.26	−0.08 (−0.24 to 0.08)	0.34
rs1211166	*NTRK2*	0.06 (−0.10 to 0.22)	0.44	−0.07 (−0.31 to 0.16)	0.54	−0.04 (−0.17 to 0.09)	0.55
rs6265	*BDNF*	0.08 (−0.10 to 0.26)	0.39	−0.12 (−0.38 to 0.14)	0.36	−0.15 (−0.30 to 0.04)	0.14
rs1401635	*BDNF*	0.08 (−0.07 to 0.24)	0.30	0.18 (−0.04 to 0.41)	0.11	0.06 (−0.06 to 0.19)	0.33
rs997295	*MAP2K5*	−0.06 (−0.08 to 0.19)	0.43	−0.05 (−0.24 to 0.15)	0.64	−0.04 (−0.15 to 0.07)	0.49
rs7203521	*FTO*	0.01 (−0.13 to 0.15)	0.86	−0.04 (−0.24 to 0.16)	0.69	−0.01 (−0.12 to 0.10)	0.86
rs1421085	*FTO*	0.40 (0.26 to 0.54)	4.4 × 10^−8^	0.54 (0.34 to 0.74)	2.2 × 10^−7^	0.08 (−0.04 to 0.19)	0.19
rs1805081	*NPC1*	0.6 (−0.09 to 0.20)	0.45	−0.10 (−0.31 to 0.10)	0.32	−0.03 (−0.15 to 0.09)	0.59
rs2075650	*APOE*	0.05 (−0.15 to 0.25)	0.63	−0.03 (−0.32 to 0.25)	0.83	0.02 (−0.14 to 0.18)	0.80
rs11671664	*GIPR*	0.22 (0.01 to 0.43)	0.04	0.36 (0.05 to 0.67)	0.02	0.15 (−0.03 to 0.32)	0.10
	GRS	0.12 (0.08 to 0.17)	2.0 × 10^−9^	0.10 (0.04 to 0.16)	1.0 × 10^−3^	−0.02 (−0.06 to 0.01)	0.21

Notes: GRS = genetic risk score.

Effect of SNPs/GRS on BMI/BAI at baseline (adjusted for gender, age, ethnicity and glycemic status).

**Table 5:** Interaction analyses between physical activity measures and obesity predisposing SNPs/GRS.

Interaction terms	SNP Main Effect			SNP Interaction		
	β	95% CI	P	β	95% CI	P
**Baseline Interaction Tests**						
**Outcome: BMI baseline**						
*FTO* rs1421085 x MET score	1.26	0.47–2.05	1.9 × 10^−5^	−0.14	−0.26–−0.01	0.03
	Subgroup analysis		Low MET tertile	0.60 kg/m^2^	0.30–0.91	1.1 × 10^−4^
			Middle MET tertile	0.42 kg/m^2^	0.15–0.69	2.5 × 10^−3^
			High MET tertile	0.26 kg/m^2^	−1.4 × 10^−3^–0.51	0.05
*TNNI3K* rs1514176 x MET score	0.38	−0.38–1.15	0.33	−0.02	−0.14− 0.10	0.72
*CDKAL1* rs2206734 x MET score	0.27	−0.69–1.23	0.58	−3.4 × 10^−3^	−0.16–0.15	0.97
*GIPR* rs11671664 xMET score	1.48	0.27–2.68	0.02	−0.18	−0.37–0.01	0.07
GRS x MET score	0.60	0.37–0.84	4.3 × 10^−7^	−0.07	−0.11–0.04	0.11
*FTO* rs1421085 x BPA score	1.08	0.69–1.47	5.4 × 10^−8^	−0.34	−0.54–−0.14	8.5 × 10^−4^
	Subgroup analysis		Low PA	0.71 kg/m^2^	0.44–0.97	1.4 × 10^−7^
			Moderate PA	0.36 kg/m^2^	0.20–0.52	9.0 × 10^−6^
			High PA	0.35 kg/m^2^	0.04–0.66	0.03
*TNNI3K* rs1514176 x BPA score	−0.17	−0.55–0.21	0.38	0.20	0.01–0.40	0.04
*CDKAL1* rs2206734 x BPA score	0.65	0.18–1.13	6.8 × 10^−3^	−0.21	−0.45–0.03	0.09
*GIPR* rs11671664 x BPA score	0.48	−0.13–1.09	0.12	−0.10	−0.41–0.21	0.52
GRS x BPA score	0.22	0.11–0.34	1.2 × 10^−4^	−0.05	−0.11–0.01	0.09
**Outcome: BAI baseline**						
*FTO* rs1421085 x MET score	1.21	0.36–2.07	5.4 × 10^−3^	−0.14	−0.28–−0.01	0.04
	Subgroup analysis		Low MET tertile	0.45	0.13–0.76	5.7 × 10^−3^
			Middle MET tertile	0.39	0.10–0.69	0.01
			High MET tertile	0.20	−0.08–0.48	0.15
*TNNI3K* rs1514176 x MET score	0.34	−0.48–1.17	0.42	−0.02	−0.15–0.11	0.73
*CDKAL1* rs2206734 x MET score	−0.10	−1.13–0.94	0.85	0.06	−0.11–0.23	0.45
*GIPR* rs11671664 x MET score	1.06	−0.24–2.35	0.11	−0.12	−0.33–0.09	0.25
GRS x MET score	0.47	0.21–0.72	2.9 × 10^−4^	−0.06	−0.10–0.02	0.07
*FTO* rs1421085 x BPA score	0.95	0.53–1.37	1.0 × 10^−5^	−0.31	−0.53 − −0.10	4.4 × 10^−3^
	Subgroup analysis		Low PA	0.62	0.34–0.91	2.1 × 10^−5^
			Moderate PA	0.25	0.08–0.42	4.9 × 10^−3^
			High PA	0.40	0.08–0.72	0.02
*TNNI3K* rs1514176 x BPA score	−0.21	−0.62–0.20	0.32	0.19	−0.02–0.40	0.08
*CDKAL1* rs2206734 x BPA score	0.57	0.06–1.08	0.03	−0.14	−0.40–0.13	0.31
*GIPR* rs11671664 x BPA score	0.34	−0.32–1.00	0.31	−0.06	−0.40–0.28	0.72
GRS x BPA score	0.19	0.07–0.31	2.7 × 10^−3^	−0.04	−0.10–0.03	0.27
**Follow-up Interaction Tests**						
**Outcome: BMI follow-up**						
*FTO* rs1421085 x MET score	1.33	0.29–2.37	0.01	−0.16	−0.33–0.01	0.06
*TNNI3K* rs1514176 x MET score	0.63	−0.38–1.64	0.22	−0.06	−0.22–0.10	0.48
*GIPR* rs11671664 x MET score	0.25	−1.41–1.91	0.77	0.01	−0.26–0.27	0.96
GRS x MET score	0.47	0.16–0.79	3.2 × 10^−3^	−0.06	−0.11–0.01	0.18
*FTO* rs1421085 x BPA score	1.10	0.59–1.61	2.3 × 10^−5^	−0.36	−0.62–−0.10	6.8 × 10^−3^
	Subgroup analysis		Low PA	0.72 kg/m^2^	0.40–1.03	1.1 × 10^−5^
			Moderate PA	0.36 kg/m^2^	0.15–0.57	7.8 × 10^−4^
*TNNI3K* rs1514176 x BPA score			High PA	0.19 kg/m^2^	−0.25–0.64	0.39
	−0.11	−0.62–0.39	0.65	0.16	−0.10–0.42	0.23
*GIPR* rs11671664 x BPA score	0.56	−0.24–1.36	0.17	−0.16	−0.57–0.24	0.43
GRS x BPA score	0.15	−7.3 × 10^−4^–0.30	0.05	−0.29	−0.11–0.05	0.47
**Outcome: BAI follow-up**						
*FTO* rs1421085 x MET score	1.91	0.69–3.14	2.3 × 10^−3^	−0.24	−0.43–0.04	0.17
*TNNI3K* rs1514176 x MET score	0.57	−0.61–1.75	0.34	−0.05	−0.23–0.14	0.64
*GIPR* rs11671664 x MET score	−0.09	−2.03–1.85	0.93	0.08	−0.24 −0.39	0.63
GRS x MET score	0.57	0.20–0.93	2.7 × 10^−3^	−0.08	−0.14–0.02	0.07
*FTO* rs1421085 x BPA score	1.22	0.60–1.84	1.2 × 10^−4^	−0.40	−0.71–−0.08	0.01
	Subgroup analysis		Low PA	0.75	0.34–1.17	3.4 × 10^−4^
			Moderate PA	0.43	0.18–0.68	6.9 × 10^−4^
			High PA	0.19	−0.29–0.67	0.44
*TNNI3K* rs1514176 x BPA score	0.18	−0.42–0.78	0.56	0.01	−0.30–0.32	0.96
*GIPR* rs11671664 x BPA score	0.75	−0.22–1.72	0.13	−0.21	−0.70–0.29	0.41
GRS x BPA score	0.21	0.03–0.39	0.02	−0.06	−0.16–0.03	0.18
**Outcome: BMI Change**						
*CDKAL1* rs2206734 x BPA score	−0.18	−0.45–0.11	0.22	0.02	−0.13–0.17	0.81
**Outcome: BAI Change**						
*CDKAL1* rs2206734 x BPA score	−0.20	−0.64–0.23	0.36	0.01	−0.21–0.24	0.92

Notes: BPA score = basic physical activity score.

GRS = Genetic risk Score.
