# Supplementary material for: Physical activity and genetic predisposition to obesity in a multiethnic longitudinal study
Source: Sci Rep. 2016 Jan 4;6:18672. doi: 10.1038/srep18672 (PMC4698633; doi:10.1038/srep18672)
Supplement: Supplementary Information [file srep18672-s1.pdf]

Physical activity and genetic predisposition to obesity in a multiethnic longitudinal study

Hudson Reddon[1], Hertzel C. Gerstein[1,2,3], James C. Engert[4], Viswanathan Mohan[5], Jackie Bosch[2], Dipika Desai[1,2], Swneke D. Bailey[4], Rafael Diaz[6], Salim Yusuf[1,2,3], Sonia S. Anand[1,2,3], David Meyre[1,2,7*]

[1]Department of Clinical Epidemiology and Biostatistics, McMaster University, Hamilton, Ontario, Canada; [2]Population Health Research Institute, McMaster University and Hamilton Health Sciences, Hamilton General Hospital, Hamilton, Ontario, Canada; [3]Department of Medicine, McMaster University, Hamilton, Ontario, Canada; [4]Departments of Medicine and Human Genetics, McGill University, Montreal, QC, Canada; [5]Madras Diabetes Research Foundation, Chennai, India; [6]ECLA—Academic Research Organization, Rosario, Argentina; [7]Department of Pathology and Molecular Medicine, McMaster University, Hamilton, Ontario, Canada;

Supplementary Table 1. Genetic information from the EpiDREAM study.

SNP information

**rs**	1514176	6235	6232	2206734	2272903	1211166	6265	1401635	997295	7203521	1421085	1805081	2075650	11671664
Gene	*TNN13K*	*PCSK1*	*PCSK1*	*CDKAL1*	*TFA2B*	*NTRK2*	*BDNF*	*BDNF*	*MAP2K5*	*FTO*	*FTO*	*NPC1*	*APOE*	*GIPR*
Risk Allele	G	C	G	C	G	A	G	C	T	A	C	A	A	G
Major Allele	A	G	A	C	G	A	G	G	T	A	T	A	A	A
Minor Allele	G	C	G	T	A	G	A	C	G	G	C	G	G	A

Frequency of Risk Allele

**rs**	1514176	6235	6232	2206734	2272903	1211166	6265	1401635	997295	7203521	1421085	1805081	2075650	11671664
**Gene**	*TNN13K*	*PCSK1*	*PCSK1*	*CDKAL1*	*TFA2B*	*NTRK2*	*BDNF*	*BDNF*	*MAP2K5*	*FTO*	*FTO*	*NPC1*	*APOE*	*GIPR*
European	0.419	0.265	0.049	0.802	0.893	0.807	0.814	0.291	0.590	0.610	0.431	0.612	0.139	0.107
North American	0.560	0.243	0.029	0.781	0.892	0.869	0.844	0.225	0.441	0.390	0.239	0.680	0.106	0.108
Latin American	0.518	0.213	0.029	0.799	0.859	0.798	0.841	0.218	0.457	0.469	0.328	0.692	0.112	0.089
East Asian	0.689	0.296	0.004	0.662	0.793	0.811	0.536	0.080	0.189	0.258	0.175	0.762	0.126	0.380
African American	0.669	0.153	0.007	0.757	0.708	0.672	0.967	0.253	0.543	0.632	0.110	0.932	0.121	0.114
South Asian	0.549	0.294	0.062	0.766	0.781	0.722	0.774	0.381	0.455	0.429	0.347	0.768	0.130	0.106

Genotype Counts

**rs**	1514176	6235	6232	2206734	2272903	1211166	6265	1401635	997295	7203521	1421085	1805081	2075650	11671664
**Gene**	*TNN13K*	*PCSK1*	*PCSK1*	*CDKAL1*	*TFA2B*	*NTRK2*	*BDNF*	*BDNF*	*MAP2K5*	*FTO*	*FTO*	*NPC1*	*APOE*	*GIPR*
European	GG,1656<br>AG,4560<br>AA,3179	GG,5092<br>CG,3617<br>CC,685	AA,8504<br>AG,868<br>GG,23	CC,6025<br>CT,3023<br>TT,346	GG,7496<br>AG,1780<br>AA,119	AA,6126<br>AG,2902<br>GG,363	GG,6237<br>AG,2812<br>AA,346	GG,4763<br>CG,3805<br>CC,826	GG,1599<br>GT,4503<br>TT,3293	GG,1474<br>AG,4379<br>AA,3542	TT,3097<br>CT,4493<br>CC,1805	AA,3555<br>AG,4396<br>GG,1444	AA,6961<br>AG,2253<br>GG,181	AA,122<br>AG,1768<br>GG,7502
North American	GG,157<br>AG,46<br>AA,97	GG,284<br>CG,189<br>CC,27	AA,471<br>AG,29<br>GG,0	CC,299<br>CT,183<br>TT,18	GG,396<br>AG,100<br>AA,4	AA,379<br>AG,111<br>GG,10	GG, 358<br>AG,128<br>AA,14	GG,303<br>CG,169<br>CC,28	GG,168<br>GT,223<br>TT,109	GG,203<br>AG,203<br>AA,93	TT,297<br>CT,167<br>CC,36	AA,224<br>AG,232<br>GG,44	AA,399<br>AG,96<br>GG,5	AA,3<br>AG,102<br>GG,395
Latin American	GG,921<br>AG,1565<br>AA,806	GG,2049<br>CG,1086<br>CC,157	AA,3104<br>AG,185<br>GG,3	CC,2107<br>CT,1049<br>TT,136	GG,2436<br>AG,784<br>AA,72	AA,2117<br>AG,1021<br>GG,154	GG, 2336<br>AG,862<br>AA,94	GG,2016<br>CG,1120<br>CC,156	GG,995<br>GT,1586<br>TT,710	GG,972<br>AG,1550<br>AA,769	TT,1513<br>CT,1396<br>CC,383	AA,1591<br>AG,1373<br>GG,328	AA,2598<br>AG,647<br>GG,47	AA,29<br>AG,532<br>GG,2730
East Asian	GG,120<br>AG,70<br>AA,35	GG,112<br>CG,93<br>CC,20	AA,223<br>AG,2<br>GG,0	CC,102<br>CT,94<br>TT,29	GG,140<br>AG,77<br>AA,8	AA,151<br>AG,63<br>GG,11	GG, 62<br>AG,117<br>AA,46	GG,193<br>CG,28<br>CC,4	GG,151<br>GT,63<br>TT,11	GG,124<br>AG,86<br>AA,15	TT,151<br>CT,69<br>CC,5	AA,133<br>AG,77<br>GG,15	AA,173<br>AG,47<br>GG,5	AA,41<br>AG,89<br>GG,95
African American	GG,560<br>AG,552<br>AA,137	GG,893<br>CG,329<br>CC,27	AA,1231<br>AG,18<br>GG,0	CC,715<br>CT,462<br>TT,72	GG,641<br>AG,487<br>AA,121	AA,568<br>AG,541<br>GG,139	GG,1171<br>AG,74<br>AA,4	GG,694<br>CG,478<br>CC,77	GG,262<br>GT,617<br>TT,370	GG,175<br>AG,570<br>AA,504	TT,986<br>CT,251<br>CC,12	AA,1085<br>AG,158<br>GG,6	AA,958<br>AG,278<br>GG,13	AA,15<br>AG,256<br>GG,978
South Asian	GG,834<br>AG,1362<br>AA,566	GG,1402<br>CG,1097<br>CC,262	AA,2437<br>AG,309<br>GG,16	CC,1628<br>CT,974<br>TT,160	GG,1683<br>AG,947<br>AA,132	AA,1440<br>AG,1106<br>GG,216	GG,1660<br>AG,956<br>AA,146	GG,1080<br>CG,1257<br>CC,424	GG,838<br>GT,1337<br>TT,587	GG,912<br>AG,1328<br>AA,521	TT,1201<br>CT,1204<br>CC,357	AA,1634<br>AG,973<br>GG,155	AA,2090<br>AG,621<br>GG,51	AA,33<br>AG,524<br>GG,2205

SNP Call Rate (%)

**rs**	1514176	6235	6232	2206734	2272903	1211166	6265	1401635	997295	7203521	1421085	1805081	2075650	11671664
**Gene**	*TNN13K*	*PCSK1*	*PCSK1*	*CDKAL1*	*TFA2B*	*NTRK2*	*BDNF*	*BDNF*	*MAP2K5*	*FTO*	*FTO*	*NPC1*	*APOE*	*GIPR*
European	100	99.989	100	100	100	99.979	100	99.989	100	100	100	100	100	99.968
North American	100	100	100	100	100	100	100	100	100	99.800	100	100	100	100
Latin American	100	100	100	100	100	100	100	100	99.970	99.970	100	100	100	99.970
East Asian	100	100	100	100	100	100	100	100	100	100	100	100	100	100
African American	100	100	100	100	100	99.920	100	100	100	100	100	100	100	100
South Asian	100	99.964	100	99.964	100	100	100	99.964	100	99.964	100	100	100	100

Hardy-Weinberg Equilibrium Values

**rs**	1514176	6235	6232	2206734	2272903	1211166	6265	1401635	997295	7203521	1421085	1805081	2075650	11671664
**Gene**	*TNN13K*	*PCSK1*	*PCSK1*	*CDKAL1*	*TFA2B*	*NTRK2*	*BDNF*	*BDNF*	*MAP2K5*	*FTO*	*FTO*	*NPC1*	*APOE*	*GIPR*
European	0.766	0.243	0.823	0.171	0.257	0.353	0.232	0.083	0.404	0.048	0.014	0.170	0.863	0.144
North American	0.978	0.627	0.987	0.113	0.492	0.553	0.496	0.520	0.037	0.002	0.067	0.151	0.989	0.247
Latin American	0.009	0.375	0.757	0.785	0.347	0.064	0.172	0.918	0.138	0.002	0.027	0.218	0.334	0.519
East Asian	$7x10^{-5}$	0.874	0.989	0.371	0.684	0.194	0.592	0.039	0.194	0.995	0.667	0.461	0.368	0.016
African American	0.997	0.742	0.977	0.817	0.033	0.519	0.035	0.652	0.954	0.542	0.365	0.820	0.184	0.673
South Asian	0.818	0.028	0.097	0.367	0.956	0.850	0.587	0.070	0.205	0.332	0.044	0.557	0.615	0.765

Supplementary Table 2. List of 41 self-reported physical activities.

1. Aerobics/Calisthenics
2. Badminton
3. Basketball
4. Bicycling
5. Bowling
6. Pilates
7. Dance
8. Fishing
9. Soccer
10. Yard work
11. Golfing
12. Hiking
13. Hockey
14. Horseback riding
15. Jogging
16. Jump rope
17. Martial arts
18. Squash
19. Mountain climbing
20. Rugby
21. Scuba diving
22. Skating
23. Snowshoeing
24. Downhill skiing
25. Cross country skiing
26. Softball
27. Stairs
28. Weightlifting
29. Swimming
30. Ping pong
31. Tai chi
32. Tennis
33. Volleyball
34. Walking
35. Aquatics exercise
36. Water skiing
37. Wood chopping
38. Work related travel (walking, cycling)
39. Work related activity (sitting, walking, carrying loads)
40. Yoga
41. Rowing

Supplementary Table 3. LD parameters between *PCSK1* rs6232 / rs6235, *BDNF* rs6265 / rs1401635, *FTO* rs1421085 / rs7203521.

**Gene**	***PCSK1* rs6232 and *PCSK1* s6235**		***BDNF* rs6265 and *BDNF* rs1401635**		***FTO* rs1421085 and *FTO* rs7203521**	
	**r2**	**D'**	**r2**	**D'**	**r2**	**D'**
**South Asian**	0.081	0.724	0.177	0.993	0.001	0.040
**East Asian**	0.006	1	0.079	1	0.004	0.084
**European**	0.125	0.938	0.093	0.998	0.062	0.359
**African**	0.037	0.921	0.012	1	0.003	0.120
**Latin American**	0.087	0.874	0.052	1	0.107	0.444
**Native North American**	0.099	1	0.051	1	0.207	0.638

Supplementary Table 4. Ethnic and country distribution of study participants.

**Country**	**Ethnic Group**						
	South Asian	East Asian	European	African	Latin American	Native North-American	**Total**
Canada	232	120	3678	44	178	479	4731
USA	26	76	787	939	337	21	2186
Bermuda	6	3	258	225	1	0	493
Germany	1	0	1132	0	0	0	1133
Netherlands	1	4	289	2	0	0	296
UK	6	0	179	1	0	0	186
Finland	0	0	356	0	0	0	356
Hungary	0	0	129	0	0	0	129
Poland	0	0	169	0	0	0	169
Slovakia	0	0	411	0	0	0	411
Turkey	0	0	442	0	0	0	442
India	2479	2	1	0	0	0	2482
Latvia	0	0	136	0	0	0	136
Australia	11	10	503	1	3	0	528
Brazil	0	9	104	37	175	0	325
Chile	0	0	1	0	378	0	379
Argentina	0	1	820	0	2220	0	3041
**Total**	2762	225	9395	1249	3292	500	17423

Supplementary Table 5. Effect of SNPs/GRS on physical activity measures.

Effect of SNPs/GRS on basic physical activity score and MET score adjusted for sex, age, ethnicity, glucose status and BAI.

SNP	Gene	OR (95% CI)[a]	P-Value[a]	β (95% CI)[b]	P-Value[b]
rs1514176	*TNNI3K*	1.02 (0.97 to 1.06)	0.48	-0.01 (-0.04 to 0.03)	0.67
rs6235	*PCSK1*	0.94 (0.89 to 1.01)	0.27	0.01 (-0.03 to 0.04)	0.76
rs6232	*PCSK1*	1.03 (0.92 to 1.15)	0.59	-0.02 (-0.10 to 0.06)	0.67
rs2206734	*CDKAL1*	1.00 (0.95 to 1.05)	0.97	-0.01 (-0.05 to 0.03)	0.51
rs2272903	*TFAP2B*	1.03 (0.97 to 1.09)	0.37	0.01 (-0.04 to 0.05)	0.71
rs1211166	*NTRK2*	1.07 (1.01 to 1.12)	0.02	-4.0×10^{-3} (-0.04 to 0.03)	0.84
rs6265	*BDNF*	1.05 (0.99 to 1.12)	0.09	0.04 (-0.01 to 0.08)	0.11
rs1401635	*BDNF*	1.07 (1.02 to 1.13)	6.4×10^{-3}	-0.02 (-0.05 to 0.02)	0.42
rs997295	*MAP2K5*	1.03 (0.99 to 1.08)	0.16	3.3×10^{-3} (-0.03 to 0.04)	0.84
rs7203521	*FTO*	1.03 (0.98 to 1.07)	0.28	-0.01 (-0.04 to 0.02)	0.57
rs1421085	*FTO*	0.96 (0.92 to 1.01)	0.08	0.01 (-0.02 to 0.04)	0.56
rs1805081	*NPC1*	0.92 (0.88 to 0.97)	1.1×10^{-3}	-0.01 (-0.04 to 0.02)	0.57
rs2075650	*APOE*	0.96 (0.90 to 1.03)	0.24	-0.02 (-0.07 to 0.02)	0.31
rs11671664	*GIPR*	1.04 (0.97 to 1.12)	0.26	0.01 (-0.05 to 0.05)	0.85
	GRS	1.01 (0.99 to 1.02)	0.23	-1.3×10^{-3} (-0.01 to 0.01)	0.80

Notes: GRS = genetic risk score,

[a] indicates analyses with the basic physical activity score as the outcome variable

[b] indicates analyses with the MET score as the outcome variable

Supplementary Table 6. Effect of SNPs/GRS on change in physical activity measures.
Effect of SNPs/GRS on change in basic physical activity score and MET score adjusted for sex, age, ethnicity, glucose status and BMI.

**SNP**	**Gene**	**OR (95% CI)[a]**	**P-Value[a]**	**β (95% CI)[b]**	**P-Value[b]**
rs1514176	*TNNI3K*	0.96 (0.89 to 1.03)	0.21	-0.03 (-0.07 to 0.02)	0.26
rs6235	*PCSK1*	0.92 (0.84 to 1.01)	0.06	-0.02 (-0.07 to 0.03)	0.47
rs6232	*PCSK1*	1.09 (0.90 to 1.06)	0.38	0.01 (-0.11 to 0.13)	0.92
rs2206734	*CDKAL1*	1.06 (0.97 to 1.16)	0.20	0.01 (-0.05 to 0.06)	0.82
rs2272903	*TFAP2B*	0.99 (0.90 to 1.10)	0.95	0.05 (-0.02 to 0.11)	0.18
rs1211166	*NTRK2*	0.88 (0.80 to 0.96)	$3.1x10^{-3}$	-0.03 (-0.08 to 0.03)	0.38
rs6265	*BDNF*	0.99 (0.90 to 1.10)	0.92	$2.6x10^{-3}$ (-0.06 to 0.07)	0.92
rs1401635	*BDNF*	0.94 (0.87 to 1.03)	0.18	-0.03 (-0.08 to 0.03)	0.30
rs997295	*MAP2K5*	1.07 (0.99 to 1.15)	0.06	$-4.7x10^{-4}$ (-0.05 to 0.05)	0.98
rs7203521	*FTO*	1.01 (0.94 to 1.08)	0.88	-0.04 (-0.09 to 0.01)	0.10
rs1421085	*FTO*	1.04 (0.98 to 1.06)	0.15	-0.03 (-0.08 to 0.01)	0.17
rs1805081	*NPC1*	1.02 (0.94 to 1.10)	0.69	$-1.3x10^{-3}$ (-0.05 to 0.05)	0.98
rs2075650	*APOE*	1.03 (0.92 to 1.14)	0.65	-0.05 (-0.11 to 0.02)	0.19
rs11671664	*GIPR*	1.09 (0.97 to 1.23)	0.14	0.03 (-0.05 to 0.10)	0.51
	GRS	1.06 (0.86 to 1.16)	0.58	-0.01 (-0.03 to $3.6x10^{-4}$)	0.06

Notes: GRS = genetic risk score,
[a] indicates analyses with the basic physical activity score as the outcome variable
[b] indicates analyses with the MET score as the outcome variable

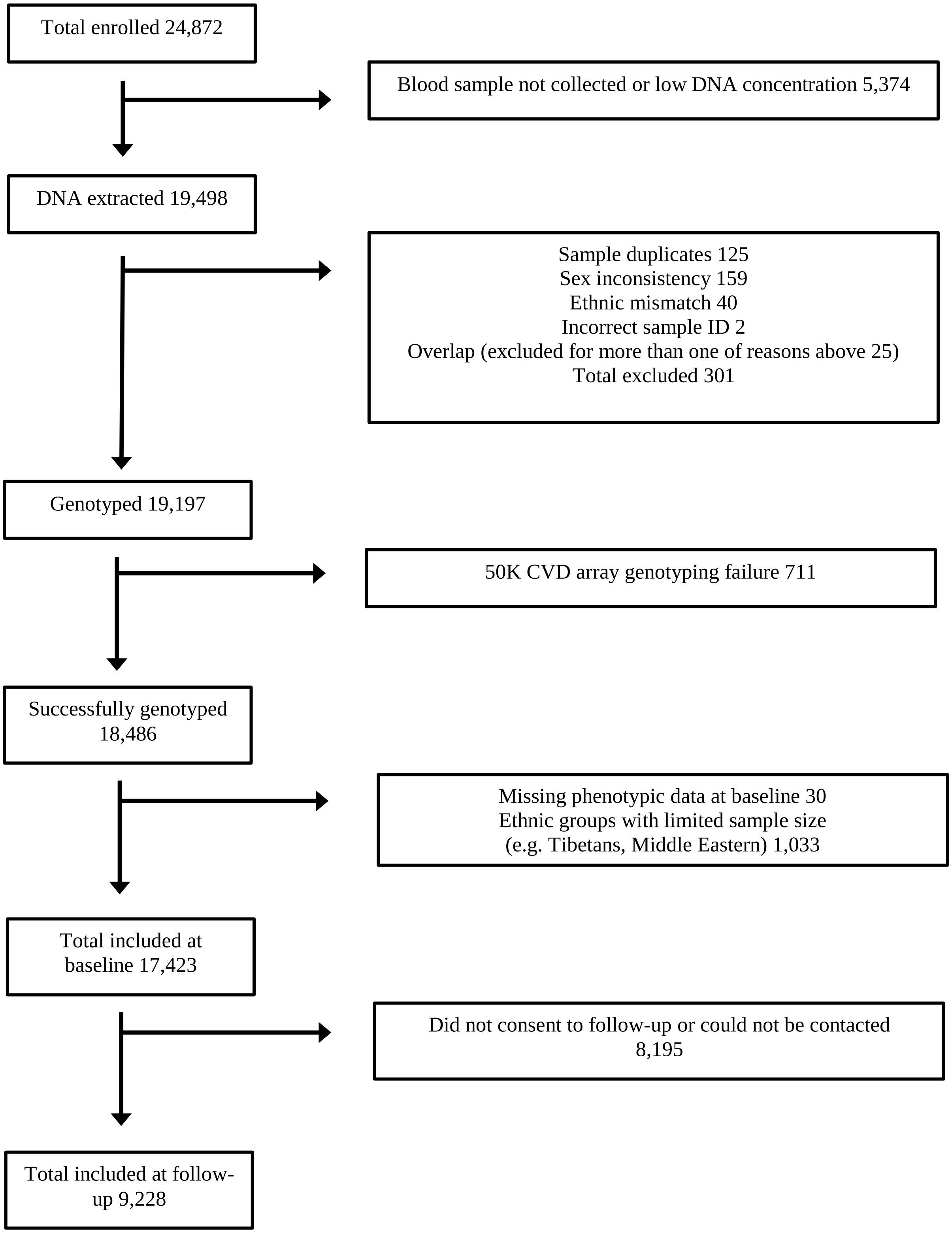

Supplementary Figure 1. Flow chart of EpiDREAM study.

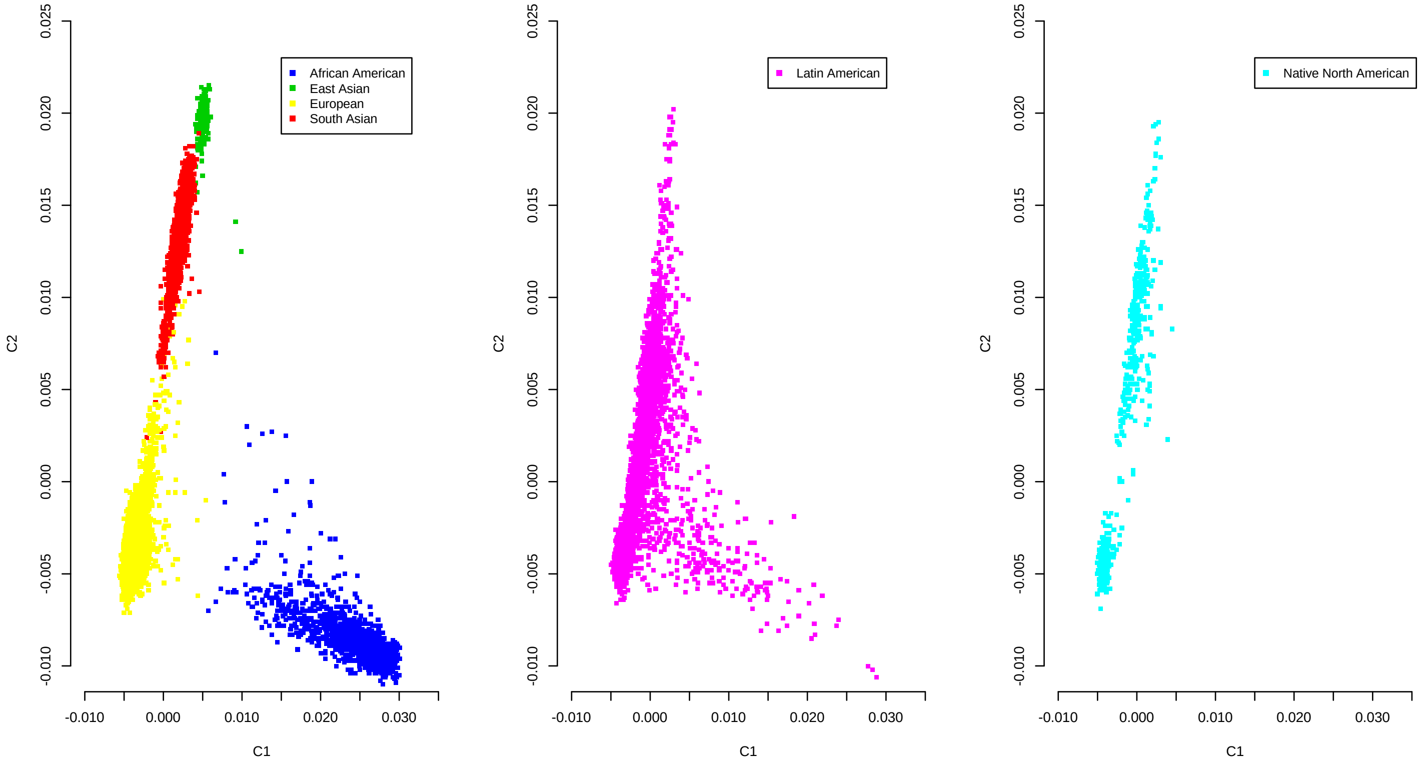

Supplementary Figure 2. Principal component analysis plot of ethnicity analysis in the EpiDREAM study.

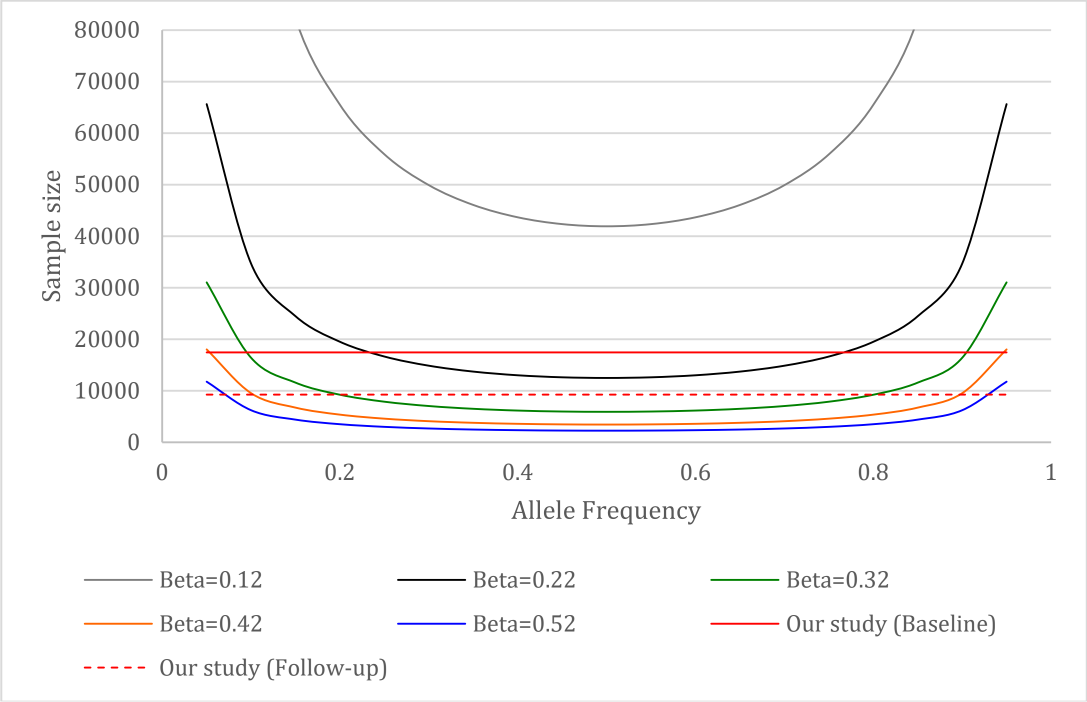

Supplementary Figure 3. Power calculation for main effect of obesity predisposing SNPs on BMI for a 2 sided P-value=0.05, 80% power (unadjusted for the multiple testing)**.**

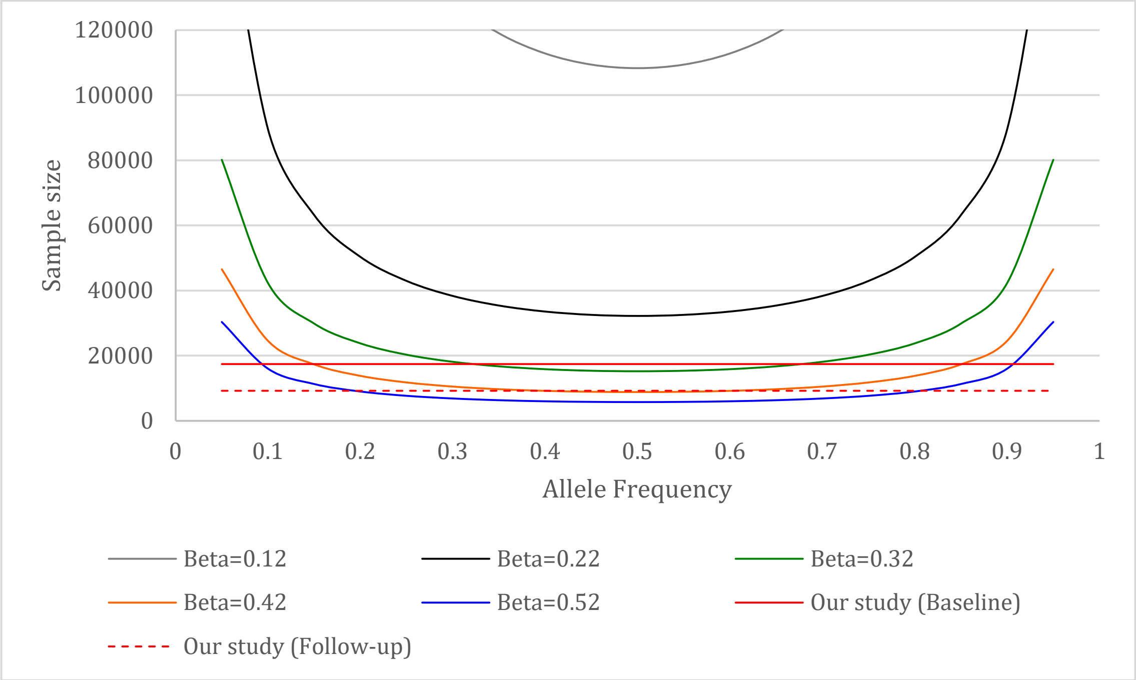

Supplementary Figure 4. Power calculation for main effect of obesity predisposing SNPs for a 2 sided P-value=0.00025, 80% power (adjusted for the multiple testing)**.**

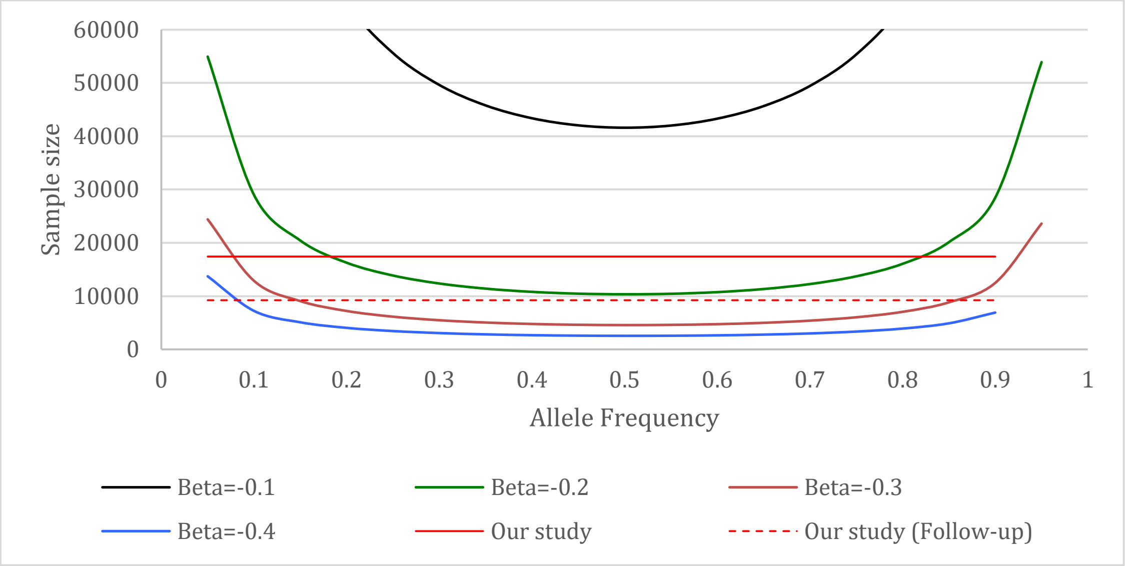

Supplementary Figure 5. Power calculation for the interaction effect between obesity predisposing SNPs and physical activity for a 2 sided P-value=0.05, 80% power (unadjusted for the multiple testing).

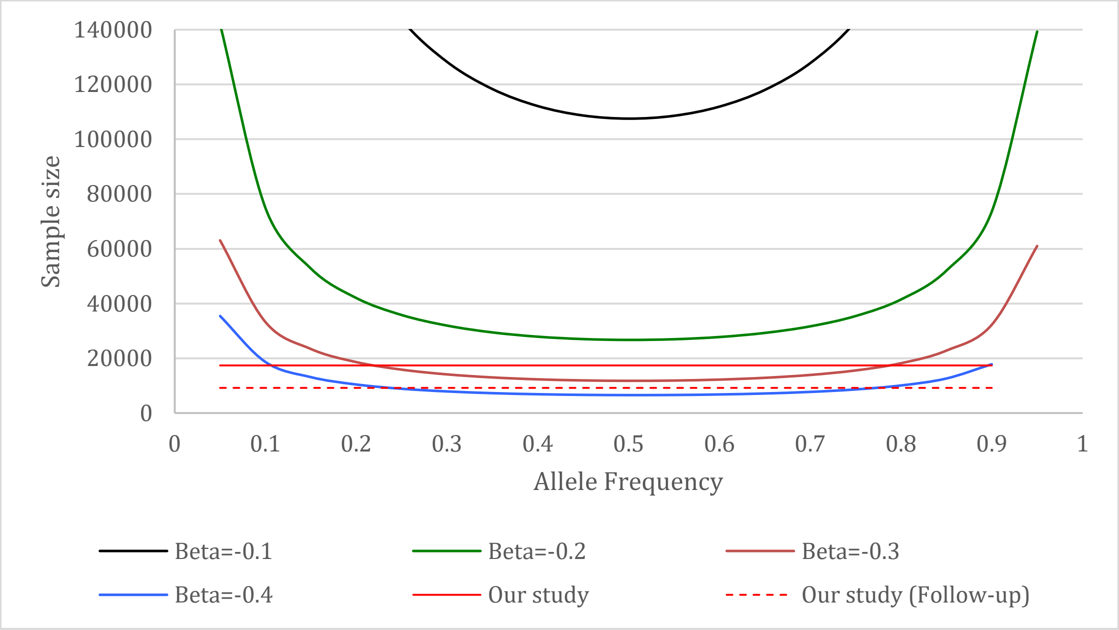

Supplementary Figure 6. Power calculation for the interaction effect between obesity predisposing SNPs and physical activity for a 2 sided P-value=0.00025, 80% power (adjusted for the multiple testing).
